# The draft genome assembly of the cosmopolitan pelagic fish dolphinfish *Coryphaena hippurus*

**DOI:** 10.1093/g3journal/jkaf059

**Published:** 2025-03-19

**Authors:** Silvia Hinojosa-Alvarez, Verónica Mendoza-Portillo, Rocío Alejandra Chavez-Santoscoy, Jesús Hernández-Pérez, Andrea Felix-Ceniceros, Erika Magallón-Gayón, Adan Fernando Mar-Silva, Maried Ochoa-Zavala, Píndaro Díaz-Jaimes

**Affiliations:** Tecnologico de Monterrey, Escuela de Ingenieria y Ciencias, Campus Monterrey, Ave. Eugenio Garza Sada 2501 Sur, Monterrey 64849, México; Unidad Académica de Ecología y Biodiversidad Acuática, Instituto de Ciencias del Mar y Limnología, Universidad Nacional Autónoma de México, Circuito Interior s/n, Ciudad Universitaria, Ciudad de México, C.P. 04510, México; Tecnologico de Monterrey, Escuela de Ingenieria y Ciencias, Campus Monterrey, Ave. Eugenio Garza Sada 2501 Sur, Monterrey 64849, México; Tecnologico de Monterrey, Escuela de Ingenieria y Ciencias, Campus Monterrey, Ave. Eugenio Garza Sada 2501 Sur, Monterrey 64849, México; Tecnologico de Monterrey, Escuela de Ingenieria y Ciencias, Campus Monterrey, Ave. Eugenio Garza Sada 2501 Sur, Monterrey 64849, México; Tecnologico de Monterrey, Escuela de Ingenieria y Ciencias, Campus Monterrey, Ave. Eugenio Garza Sada 2501 Sur, Monterrey 64849, México; Unidad Académica de Ecología y Biodiversidad Acuática, Instituto de Ciencias del Mar y Limnología, Universidad Nacional Autónoma de México, Circuito Interior s/n, Ciudad Universitaria, Ciudad de México, C.P. 04510, México; Escuela Nacional de Estudios Superiores, Unidad Morelia, Universidad Nacional Autónoma de México, Antigua Carretera a Pátzcuaro No. 8701. Col. Ex Hacienda de San José de la Huerta. Morelia, Michoacán C.P. 58190, México; Unidad Académica de Ecología y Biodiversidad Acuática, Instituto de Ciencias del Mar y Limnología, Universidad Nacional Autónoma de México, Circuito Interior s/n, Ciudad Universitaria, Ciudad de México, C.P. 04510, México

**Keywords:** Coryphaenidae, dolphinfish, nanopore, comparative genomics, genome assembly, de novo assembly

## Abstract

For the first time, the complete genome assembly of the dolphinfish (*Coryphaena hippurus*), a tropical cosmopolitan species with commercial fishing importance was sequenced. Using a combination of Illumina and Nanopore sequencing technologies, a draft genome of 497.8 Mb was assembled into 6,044 contigs, with an N50 of 200.9 kb and a BUSCO genome completeness score of 89%. This high-quality genome assembly provides a valuable resource to study adaptive evolutionary processes and supports conservation and management strategies for this ecologically and economically significant species.

## Introduction

The dolphinfish (*Coryphaena hippurus*, family Coryphaenidae, order Carangiformes) is a widely distributed pelagic species found in tropical and subtropical waters globally (Farrell *et al*. 2014). Known for its rapid growth rate, short lifespan, and high fecundity, this species plays a critical role in marine food webs, acting as both a predator and prey (Oxenford 1999). Additionally, *C. hippurus* supports significant commercial and recreational fisheries, making it economically valuable and an important target for sustainable management practices (Oxenford 1999). Its broad habitat range, spanning multiple environmental gradients, and high dispersal capacity make it an excellent model for studying the genetic mechanisms of adaptation and population differentiation across diverse oceanic conditions.

Sequencing the genome of *C. hippurus* is crucial to understanding the genetic basis of its adaptations to environmental variability, including temperature, salinity, and food availability. Such insights are especially important given the increasing impact of climate change and commercial exploitation on marine biodiversity (Reiss *et al*. 2009; Pinsky and Palumbi 2014). A reference genome provides a foundational resource for identifying adaptive variants that contribute to resilience in changing environments, as well as for monitoring population health and genetic diversity over time. Furthermore, genomic data from *C. hippurus* can facilitate comparative studies within the order Carangiformes and across other ecologically and economically important fish species (Manel and Holderegger 2013; Riginos and Liggins 2013).

This study aims to produce the first genome assembly for *C. hippurus* using a combination of short-read and long-read sequencing technologies to capture the full genetic complexity of the species. Previous studies have shown that combining these technologies can enhance the quality of reference genomes for various fish species, providing comprehensive tools for population and conservation genomics (Austin *et al*. 2017; Tan *et al*. 2018; Johnson *et al*. 2020; Arias *et al*. 2021). With <1% of actinopterygian species having annotated genomes and limited genomic resources for Carangiformes, this genome will be an invaluable addition to existing resources, supporting both evolutionary studies and conservation efforts (Fan *et al*. 2020).

The genome assembly for *C. hippurus*, will enable researchers to explore genetic, ecological, and evolutionary issues central to understanding adaptation in marine species. This resource will serve also as a model for studying the effects of natural and anthropogenic pressures on marine fish populations, contributing to the sustainable management and conservation of marine biodiversity.

## Methods

### Sample collection

Specimens of *C. hippurus* were collected from Mazatlan, Mexico through commercial fisheries. A healthy adult fish was selected for genomic analysis, kept in dry ice while transporting it to the laboratory and then preserved at −80°C until further processing.

### DNA extraction

Total genomic DNA was extracted from fin tissue using the DNeasy Blood and Tissue Kit (Qiagen) following the manufacturer's protocol with slight modifications. Tissue samples were resuspended in 180 µL of ATL buffer and 20 µL of proteinase K. The homogenate was incubated at 56°C for 24 h with intermittent vortex. After digestion, 200 µL of AL buffer was added, mixed, and incubated at room temperature for 10 min. The DNA was purified using DNeasy spin columns and eluted twice in 100 µL of AE buffer at 1000×g for 1 min and 8000×g for another 1 min. DNA concentration was assessed using a Qubit dsDNA HS quantification assay kit (Thermo Fisher Scientific), purity was evaluated using a NanoDrop One (Thermo Fisher Scientific), and DNA integrity was determined through agarose gel electrophoresis. DNA samples with a concentration >100 ng/µL and that showed absence of DNA degradation in agarose gel electrophoresis were selected for sequencing.

### Illumina short-read sequencing

A genomic DNA library for Illumina sequencing was prepared using the Illumina DNA Library Prep Kit (Illumina). The library was prepared following the manufacturer's protocol. The library was quantified using a Qubit dsDNA HS quantification assay kit, and its size distribution was verified through capillary electrophoresis using a S2 DNA standard cartridge in a Qsep400 (BiOptic). Sequencing was performed on an Illumina NovaSeq 6000 platform using a 2 × 150 bp paired-end configuration.

### Nanopore long-read sequencing

Twenty-four genomic DNA libraries for Nanopore sequencing were prepared from the same genomic DNA aliquot using the Rapid Sequencing Kit V14 (Oxford Nanopore Technologies). 200 ng of high-quality DNA were used to prepare each library according to manufacturer's instructions. Final libraries were quantified using a Qubit dsDNA HS quantification assay kit. Sequencing was performed in a MinION Mk1C device (Oxford Nanopore Technologies) using one MinION flow cell per 6 libraries. Each sequencing run was performed using a new flow cell; 6 MinION flow cells were used to sequence all 36 libraries.

### Data quality control

Illumina short-read data quality from RNA and DNA was assessed using FastQC v0.11.9 (https://www.bioinformatics.babraham.ac.uk/projects/fastqc/), and reads were trimmed with Trimmomatic v0.39 (Bolger *et al*. 2014) to remove adapter sequences and low-quality bases. Nanopore long-read data quality was evaluated using Guppy v5.0.14 (https://community.nanoporetech.com/docs/prepare/library_prep_protocols/Guppy-protocol/v/gpb_2003_v1_revax_14dec2018/guppy-software-overview) for base-calling and NanoPlot v1.36.0 (https://github.com/wdecoster/NanoPlot) for read length and quality metrics. Long-read quality was checked with LongQC (Fukasawa *et al*. 2020), and remaining adapters were trimmed with Porechop (Bonenfant *et al*. 2023).

### Genome assembly

The initial assembly was performed with long reads to generate a draft genome assembly with Flye v 2.9.3 (Kolmogorov *et al*. 2019) using default parameters. The draft assembly was polished 3 rounds using Illumina short-read data with Pilon v1.24 (Walker *et al*. 2014) to correct errors, both programs were run on the Omics Box suite (BioBam Bioinformatics, https://www.biobam.com/omicsbox) with default parameters.

### Assembly evaluation

The quality and completeness of the final genome assembly were evaluated using the Benchmarking Universal Single-Copy Orthologs (BUSCO) tool v5.5.0 (Manni *et al*. 2021) with the Actinopterygii_odb10 database for genome completeness assessment run on the Omics Box suite under predetermined parameters (https://www.biobam.com/omicsbox). Seven genomes of phylogenetic related species *Perca fluviatilis*, *P. flavescens, Seriola aureovittata, S. dorsalis, Lates calcarifer, L. japonicus,* and *Caranx melampygus* were downloaded from NCBI (Table 1 and Supplementary Table 1) to compare BUSCO statistics. Assembly metrics, including N50, contig length, and the number of contigs, were analyzed using QUAST v5.2.0 (Gurevich *et al*. 2013) run under default parameters on the Omics Box suite (BioBam Bioinformatics, https://www.biobam.com/omicsbox).

**Table 1.** Genomes comparison of closely related species to *C. hippurus*.

Species	Complete BUSCOs	Single copy BUSCOs	Duplicated BUSCOs	Fragmented BUSCOs	Missing BUSCOs	N50 contigs
*C. hippurus* ^a^	88.9%	87.9%	1.0%	2.1%	9.0%	128.5 KB
*P. fluviatilis* ^b^ GCA_010015445.1 (Ozerov *et al*. 2018)	97.7%	96.9%	0.8%	0.7%	1.6%	4 MB
*P. flavescens* ^b^ GCA_004354835.1 (Feron *et al*. 2020)	3592	3556	36	13	35	4 MB
*S. aureovittata* ^b^ GCA_021018895.1 (Li *et al*. 2022a)	97.7%	96.9%	0.8%	0.7%	1.6%	17 MB
*S. dorsalis* ^c^ GCA_002814215.1 (Li *et al*. 2022b)	84.0%	83.2%	0.8%	6.0%	10.0%	11 KB
*L. japonicus* ^c^ GCA_033238685.1 (Hashiguchi *et al*. 2024)	94.5%	90.1%	4.4%	2.5%	3.0%	285 KB
*L. calcarifer* ^b^ GCA_001640805.2 (Vij *et al*. 2016)	98.7%	95.3%	3.4%	0.4%	0.9%	1 MB
*C. melampygus* ^c^ GCA_019059645.1 (Pickett *et al*. 2021)	97.8%	89.9%	7.9%	0.7%	1.5%	1 MB

^a^This study ^b^reference genome (chromosome level) ^c^scaffold.

#### Gene annotation

To improve the gene prediction RNA-Seq data was obtained from dolphinfish tissue control samples (nonoil exposed) of BioProject PRJNA763937 (Schlenker *et al*. 2022), all the control samples were concatenated and then assembled with Trinity v2.15.1 with the following command Trinty –seqType fq –single/user/dir/*.fastq.gz –CPU 30 –max_memory 150G –output/user/dir/out (Grabherr *et al*. 2011). Ab initio prediction of genes was done using the RepeatMasker, (Smit *et al.* 2013) AUGUSTUS (Stanke *et al*. 2006) and SNAP (Korf 2004). Final annotation was performed with the assembled transcripts, the vertebrate proteins database, and the ab initio predicated genes using the MAKER pipeline v3.01.03 (https://hpc.nih.gov/apps/MAKER.html). Functional annotation of predicted genes was performed using BLAST v2.11.0 against the UniProt and NCBI databases, and InterProScan v5.53-87.0 (https://www.ebi.ac.uk/interpro/search/sequence/) for domain identification run the Omics Box suite (BioBam Bioinformatics, https://www.biobam.com/omicsbox) using defaults values. A total of 4 rounds of maker were evaluated. The scripts used for MAKER pipeline can be found in https://github.com/Tec-BASE-Laboratory/MAKER_Chippurus.

### Comparative genomics

Protein sequences from the same 7 species were retrieved from the NCBI database. Additionally, protein sequences predicted by AUGUSTUS from the draft genome of *C. hyppurus* were incorporated into the analysis. Comparative genomic analysis was performed using OrthoFinder v2.5.4 (Emms and Kelly 2015) to identify orthologous gene families across the species. In addition, OrthoVenn3 web page (https://orthovenn3.bioinfotoolkits.net/) was used to construct the phylogenetic tree using single-copy orthologues and produce the graphics for orthologous cluster comparison.

## Results and discussion

### Genome assembly and quality assessment

The final genome assembly comprised 497.8 mega base pairs (Mb), with an N50 of 200.9 kb (Table 1). The largest contig measured 590,344 bp, while the shortest contig was 5,963 bp. The estimated genome size produced with findGSE (Sun *et al*. 2018) was 534,132 Mb. BUSCO v5.5.0 analysis using the actinopterygii_odb10 core gene set revealed that 89.0% of the reference dataset sequences had a complete ortholog, and 88.0% of the genes were complete single-copy genes. While the draft genome of *C. hippurus* shows feasibility for refinement compared with other genome assemblies of closely related species, it provides a robust and reliable resource for genomic studies.

### Genome annotation

#### Transcriptome assembly

**Fig. 1.**
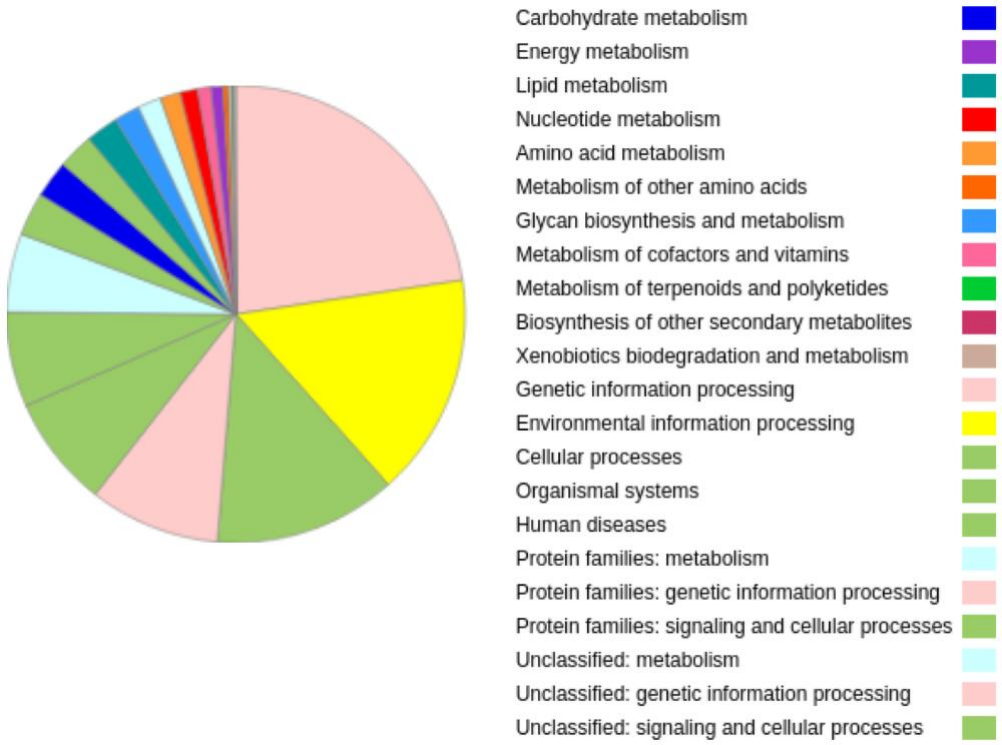
Functional classification of biological pathways in the *C. hippurus* transcriptome.

The BUSCO analysis of the transcriptome assembly indicated that 57.09% of orthologs were present as single copies, while 10% were identified as duplicated, which could reflect biological duplications or technical redundancies, such as splice variants. This analysis highlights the completeness of the transcriptome and its utility in annotating the genome. These results are consistent with the proteins obtained from AUGUSTUS on the assembled transcriptome revealed pathways primarily related to metabolism, environmental processing, and cellular processing (Fig. 1).

### Annotation

The results obtained with Repeat Masker (Table 2) showed that retroelements comprise 26,987 elements spanning 3,895,527 bp, accounting for 0.78% of the total sequence. This category includes subtypes like short interspersed nuclear elements (SINEs), interspersed nuclear elements (LINEs), and long terminal repeat elements (LTR), with LINEs being the most prevalent, occupying 1,590,131 bp (0.32%). DNA transposons are more abundant, with 62,971 elements covering 7,317,893 bp, representing 1.47% of the sequence. Additionally, simple repeats are notably prominent, consisting of 310,761 elements and covering 14,556,967 bp, which corresponds to 2.93% of the sequence. The diversity and extent of repetitive sequences provide a clear overview of the genomic composition in terms of repeat content. These results are congruent with those reported for other marine fishes such as King Angelfish (*Holacanthus passer,* e.g. 1.36% of DNA tranposons, 2.14% of simple repeats, Gatins *et al*. 2024) but low in LINEs in comparation with other Carangiforms, for example, Carangidae family members, (Coryphaenidae's sister family) such as *C. melampygus*, which shows values of 2.3% for LINEs and 5.4% for DNA transposons (Pickett *et al*. 2021), or *Seriola* species (*S. lalandi* 3.85%, Li *et al*. 2022a; *S. aureovittata* 4.71% of LINEs, Li *et al*. 2022b).

**Table 2.** Summary of repetitive elements in the *C. hippurus* genome.

	Number of elements^a^	Length occupied	Percentage of sequence
Retroelements	26,987	3,895,527 bp	0.78%
SINEs:	3127	316,648 bp	0.06%
Penelope:	0	0 bp	0.00%
LINEs:	8078	1,590,131 bp	0.32%
CRE/SLACS	0	0 bp	0.00%
L2/CR1/Rex	3883	574,723 bp	0.12%
R1/LOA/Jockey	157	20,673 bp	0.00%
R2/R4/NeSL	411	196,148 bp	0.04%
RTE/Bov-B	1208	442,619 bp	0.09%
L1/CIN4	1604	289,200 bp	0.06%
LTR elements:	15,782	1,988,748 bp	0.40%
BEL/Pao	806	325,022 bp	0.07%
Ty1/Copia	14	6115 bp	0.00%
Gypsy/DIRS1	7075	916,350 bp	0.18%
Retroviral	4838	493,398 bp	0.10%
DNA transposons	62,971	7,317,893 bp	1.47%
hobo-Activator	15,409	1,390,496 bp	0.28%
Tc1-IS630-Pogo	11,908	3,291,932bp	0.66%
En-Spm	0	0 bp	0.00%
MULE-MuDR	851	48,507 bp	0.01%
PiggyBac	767	113,574 bp	0.02%
Tourist/Harbinger	1757	137,063 bp	0.03%
Other (Mirage *P*-element Transib)	1014	108,422 bp	0.02%
Rolling-circles	772	48,901 bp	0.01%
Unclassified:	173	48,350 bp	0.01%
Total interspersed repeats:		11,261,770bp	2.26%
Small RNA:	1146	100,962 bp	0.02%
Satellites:	619	58,851 bp	0.01%
Simple repeats:	310,761	14,556,967 bp	2.93%
Low complexity:	36,791	2,061,291 bp	0.41%

For each category, the number of elements detected, the total length occupied (in base pairs), and the percentage of the sequence length are provided.

### Comparative genomics

After completing 4 annotation rounds with MAKER, it was observed that round 1 and round 4 showed more gene models 34,129 and 35,670 respectively also higher annotation edit distance with 98 and 96% confidence in the gene models (Supplementary Table 1). Since round 4 had more gene models and good confidence in the gene models this was used as the final annotation.

**Fig. 2.**
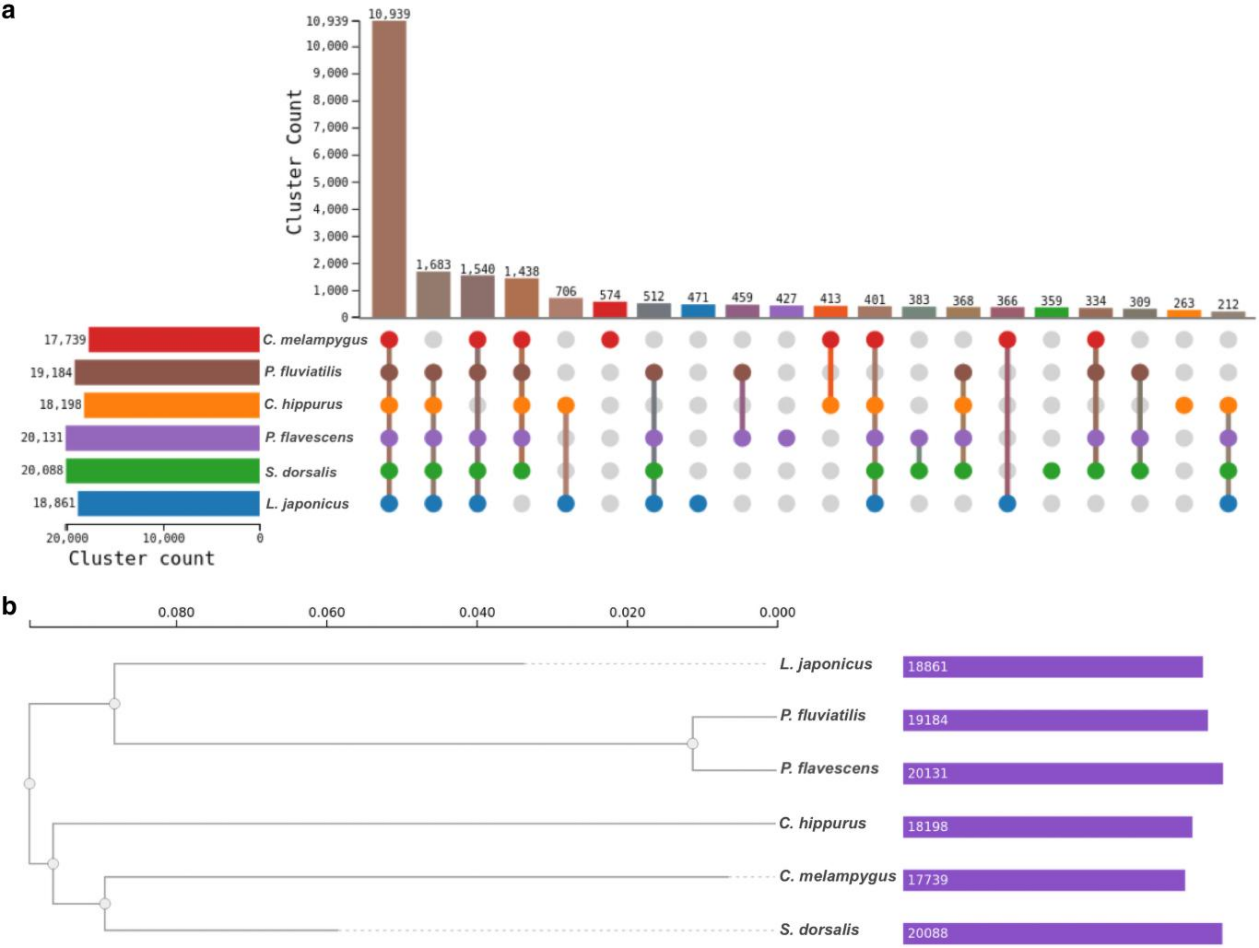
Comparative genomics of *C. hippurus* with related species. a) This figure shows the identification of orthologous gene clusters across 7 species. The number of shared and unique gene clusters is visualized. b) Phylogenetic tree constructed using single-copy orthologues, providing insights into the evolutionary relationships of *C. hippuru*s within Carangiformes.

Figure 2a visualizes the intersections of orthologous gene clusters across the 7 species, with *P. flavescens* having the highest count of 20,131 clusters and *L. japonicus* the lowest with 18,861 clusters. The main bar chart at the top shows the size of the intersections between these species’ gene clusters. The largest intersection, containing 10,939 clusters, is shared among all 7 species, indicating a significant core set of orthologous genes. Other notable intersections include smaller subsets shared among different combinations of species, as indicated by the connected dots below the bar chart. For instance, there are 1,683 clusters unique to *P. fluviatilis* and *C. melampygus*, and 1,540 clusters unique to *C. hipurus* and *P. flavescens*. The phylogenetic tree constructed with single-copy orthologues (Fig. 2b) is consistent with the previously described for Carangiformes (Glass *et al*. 2023).

**Fig. 3.**
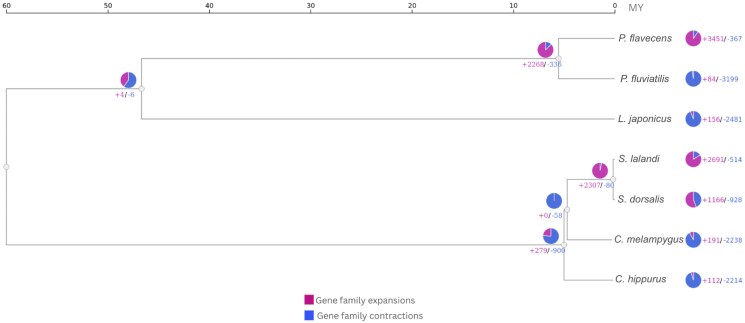
Gene family expansions (pink) and contractions (blue) in *C. hippurus* and related species. The figure represents gene family dynamics, showing expansions and contractions across species.

Gene expansions and contractions are evolutionary processes where gene families either increase or decrease in size. These changes can lead to adaptations, with expansions often providing new functions or enhancing existing ones, while contractions may reflect the loss of redundant or nonessential functions as species evolve and adapt to new environments. In Fig. 3, *P. flavecens* exhibits a significant number of gene family expansions (+3,451) compared with contractions (−367), while *P. fluviatilis* shows fewer expansions (+84) and a substantial number of contractions (−3,199). *L. japonicus* and *C. melampygus* both display moderate expansions (+156 and +191, respectively) with a higher number of contractions (−2,481 and –2,238, respectively). *S. lalandi* and *S. dorsalis* exhibit high and moderate gene family expansions (+2,691 and +1,166, respectively) and fewer contractions (−514 and −928, respectively). Meanwhile, *C. hippurus* shows a more balanced pattern with +112 expansions and –2,214 contractions. The internal nodes of the tree reflect the evolutionary history of these species, with varying degrees of expansions and contractions, such as +2,268/−336 at 1 node and +2,307/−80 at another. Overall, the analysis indicates a trend where gene family contractions often outnumber expansions, particularly in species like *P. fluviatilis*, *L. japonicus*, *C. melampygus*, and *C. hippurus*, while *P. flavecens* and *S. lalandi* show more pronounced gene family expansions. It is worth mentioning that *C. hippurus* genome is a first version and these missing regions may contribute to false gene contractions, highlighting the need for caution in interpreting gene family dynamics. Despite these missing regions, this genome provides a valuable resource for studying the genetic basis of adaptation, population structure, and conservation in a species of significant ecological and commercial importance.

*Coryphaena hippurus* presented 263 clusters unique to the species, for these gene clusters, gene ontology (GO, Ashburner *et al*. 2000) enrichment was calculated, and the enriched pathways are mainly related to biological processes (Table 3). As an example, Wnt signaling pathways are involved in the development of tissues and organs in embryos, and they are controlled by an interplay of several signaling pathways that crosstalk to provide positional information and induce cell fate specification (Huelsken and Birchmeier 2001).

**Table 3.** Enriched pathway in single copy unique orthologues of *C. hippurus*.

GO-ID	Count	Pathway	GO-therm	*P*-value
GO:0016021	2	Integral component of membrane	Cellular component	1.23E−41
GO:0003964	20	RNA-directed DNA polymerase activity	Molecular function	9.01E−26
GO:0006355	2	Regulation of transcription, DNA-templated	Biological process	5.80E−24
GO:0016055	3	Wnt signaling pathway	Biological process	2.71E−19
GO:0007283	2	Spermatogenesis	Biological process	2.48E−16
GO:0016032	2	Viral process	Biological process	1.57E−15
GO:0016192	2	Vesicle-mediated transport	Biological process	7.67E−15
GO:0046872	3	Metal ion binding	Molecular function	4.75E−14
GO:0016567	2	Protein ubiquitination	Biological process	5.87E−11
GO:0032197	8	Transposition, RNA-mediated	Biological process	1.80E−09
GO:0051056	2	Regulation of small GTPase mediated signal transduction	Biological process	0.006397882811536
GO:0006334	2	Nucleosome assembly	Biological process	0.021287316663045

## Conclusions

We present the first genome of the dolphinfish (*C. hippurus*), combining Illumina and Nanopore sequencing technologies. By using a combination of long and short-read sequencing platforms, we successfully assembled a draft genome of a nonmodel species, obtaining 89% of complete orthologous genes, which indicates that the assembly captures a significant portion of the genome's coding and functional elements. This is critical for downstream applications such as comparative genomics, evolutionary studies, and functional annotation. The assembled genome size of 0.49 Gb closely approximates the estimated size of 0.53 Gb, suggesting minimal loss of genomic information during the assembly process. This level of completeness is sufficient to provide a reliable resource to understand the molecular mechanisms underlying the acclimatization and adaptation to both local and global environmental changes of marine species, including dolphinfish, across its distributional range. These insights will be valuable for future studies on evolutionary biology, population genomics, and the design of conservation strategies for *C. hippurus*, and other nonmodel marine species from which scarce information exists, particularly in the face of accelerating climate change.

## Supplementary Material

jkaf059_Supplementary_Data

## Data Availability

Raw sequencing data and the draft genome assembly are available under the project accession number PRJNA1146410 of GenBank. Additional Supplemental material available at GSA FigShare: https://doi.org/10.25387/g3.28548704. Supplemental material available at G3 online.
